# A poly (glycerol-sebacate-acrylate) nanosphere enhanced injectable hydrogel for wound treatment

**DOI:** 10.3389/fbioe.2022.1091122

**Published:** 2023-01-12

**Authors:** Jiajia Luo, Fenglei Sun, Pinhua Rao, Tonghe Zhu, Yonghang Liu, Juan Du, Sihao Chen, Xiangyun Jin, Jiale Jin, Yi Chai

**Affiliations:** ^1^ School of Chemistry and Chemical Engineering, Institute for Frontier Medical Technology, Shanghai Engineering Research Center of Pharmaceutical Intelligent Equipment, Shanghai Frontiers Science Research Center for Druggability of Cardiovascular Non-coding RNA, Shanghai University of Engineering Science, Shanghai, China; ^2^ Department of Neurosurgery, Weifang People’s Hospital, Weifang, Shandong, China; ^3^ Department of Orthopaedics, Renji Hospital, School of Medicine, Shanghai Jiao Tong University, Shanghai, China; ^4^ Spine Lab, Department of Orthopaedic Surgery, The First Affiliated Hospital, Zhejiang University, Hangzhou, China; ^5^ Department of Neurosurgery, Renji Hospital, School of Medicine, Shanghai Jiao Tong University, Shanghai, China

**Keywords:** poly (glycerol-sebacate-acrylate) nanosphere, injectable hydrogel, antibacterial, vascularization, wound treatment

## Abstract

Wound repair remains a huge clinical challenge, which can cause bleeding, infection, and patient death. In our current research, a bioactive, injectable, multifunctional composite hydrogel doped with nanospheres was prepared with antibacterial and angiogenesis-promoting functions for the treatment of wounds. Amino groups in ε-polylysine (ε-EPL) undergo dynamic Schiff base reaction cross-linking with oxidized hyaluronic acid (OHA), and F127 exhibits unique temperature sensitivity to form an injectable thermosensitive hydrogel (FHE10), which can form a hydrogel to cover the wound at body temperature. Nanospheres (PNs) prepared using poly (glyceryl-sebacate-acrylate) (PGSA) were loaded into hydrogels (FHE10) for promoting wound repair. The prepared FHE10 exhibited rapid gelation, good injectable abilities, and showed resistance to the flourish of *Escherichia coli* (*E. coli*) and *Staphylococcus aureus* (*S. aureus*). *In vitro* investigations showed that FHE10 had good hemocompatibility and cytocompatibility. FHE10@PNs exhibited good proliferation, migration, and tube formation of human umbilical vein endothelial cells (HUVECs) and human foreskin fibroblasts (HFF-1). Furthermore, FHE10@PNs significantly promoted reepithelialization and collagen deposition as well as micro-vascularization compared with the use of FHE10 or PNs alone, thereby accelerating the repair of wounds. In general, this study demonstrated that the multifunctional injectable composite hydrogel showed great potential in wound treatment.

## 1 Introduction

Poly (glycerol-sebacate) (PGS) elastomer (Wang Y D et al., 2002) is obtained by polycondensation of two natural metabolic intermediates, glycerol, and sebacic acid, and have good biocompatibility and biodegradability. PGS can be used as scaffolds for cardiovascular (Rai et al., 2015), nerve (Saudi et al., 2019), cartilage (Souza et al., 2017), and bone (Piszko et al., 2021) tissue engineering, and has good applications in drug delivery systems (Hsieh et al., 2018) and wound repair (Keirouz et al., 2020). However, the traditional thermal crosslinking curing method requires long-term high-temperature reaction conditions (Wang Y D et al., 2002), and the cured product cannot be further processed, which leads to its limited application in biomedicine. PGS is modified in the presence of acrylate groups to form polyglyceryl sebacate acrylate (PGSA) (Nijst et al., 2007). PGSA has the ability of fast photocuring in the presence of photoinitiators, which not only solves the high-temperature problem of the reaction but also greatly reduces response time. PGSA also has good biocompatibility and biodegradability and can be used as a scaffold for tissue engineering such as heart and nerve, as well as the ability to enhance tissue vascular reconstruction and promote wound repair (Jiang et al., 2021).

Hyaluronic acid (HA) is a polysaccharide derived from the extracellular matrix in tissues, which is vital in angiogenesis (Kang et al., 2019) and wound healing (Wu et al., 2017). A variety of hydrogel scaffolds were prepared from hyaluronic acid for tissue regeneration and proved to be able to absorb body fluids rapidly, with good water retention, biodegradability, and biocompatibility (Rezaeeyazdi et al., 2018). Studies have shown that some peptides have obvious antibacterial properties (Sathoff et al., 2018). Shime and Sakai (Shima and Sakai, 1977) first discovered in the 1970s that *Streptomyces* Albicans can produce ε-polylysine, a natural polypeptide composed of L-lysine monomers, which has biodegradable properties, good water solubility, non-toxic, and other advantages. ε-EPL can be used in the food industry as an antibacterial agent (Ge et al., 2022), and in recent years, its application in biomedicine has gradually increased (Shukla et al., 2012), such as drug carrier (de la Torre et al., 2018), interferon inducer (Wakamoto et al., 2007), dental adhesive (Xie et al., 2019), wound dressings (Sun et al., 2020), *etc.* F127 has received more and more attention as a drug delivery system, biomaterial, and tissue engineering hydrogel thanks to its marvelous biocompatibility and temperature sensitivity (Park et al., 2006), but owing to its insufficient mechanical properties and rapid degradation, limiting its appliance in regenerative medicine. By modifying it or combining it with other substances, it can be widely used after improving the above shortcomings (Li et al., 2020). The Schiff base reaction is a chemical reaction that forms dynamic covalent imine bonds through the cross-linking of amine groups and aldehyde groups (McKay and Finn, 2014). The dynamic cross-linked network are important to form self-healing hydrogels in a mild way, which can make the self-healing hydrogels automatically restore their original structure and function after damage (Xu et al., 2019).

Whether it is disease or physical stimulation, it may cause skin damage, and even affect the life and health of patients in severe cases. Therefore, the development of effective wound dressings is crucial for clinical treatment. The wound dressing should have hemostatic function, keep the tissue isolated from the external environment, prevent bacterial infection, and promote the repair of wound. The wound is easy got infected by bacteria in the procedure of wound healing which leads to the long-term failure of the wound to heal, which may lead to an inflammatory response and affect the normal function of the infected tissue (Holubová et al., 2021). Therefore, the preparation of materials with antibacterial properties that can promote wound healing is crucial. The important courses of wound healing include migration, proliferation, deposition of cell, and formation of extracellular matrix (Telgenhoff and Shroot, 2005; Jie et al., 2007), all of which require the transport of oxygen, nutrients, and cytokines with the help of blood vessels, so the building of new blood vessels in wound is crucial (Michalczyk et al., 2019). Injectable hydrogels (Xuan et al., 2021) are promising candidate materials with structures like the extracellular matrix, which can be injected to cover irregular wounds, absorb body fluids at the wound surface, and isolate the external environment.

In this study, we used HA to form oxidized hyaluronic acid (OHA) by modifying it with sodium periodate. At room temperature or physiological conditions, OHA interacts with ε-EPL through Schiff base reaction to form hydrogels. F127 is temperature-sensitive and automatically form a gel at room temperature or under physiological conditions. After mixing the above three substances uniformly, an injectable multifunctional hydrogel with temperature-sensitive properties can be formed at room temperature or under physiological conditions. PGSA was synthesized by modifying PGS with acryloyl chloride. Under the action of the photoinitiator, PGSA undergoes free radical polymerization to form nanospheres. The nanospheres were doped into the hydrogel to prepare an injectable and multifunctional wound repair hydrogel dressing for promoting angiogenesis.

## 2 Materials and methods

### 2.1 Materials

Glycerol was provided by China National Pharmaceutical Group Co. (Shanghai, China). Sebacic acid (SA), acryloyl chloride, ethyl acetate (EA), ethanol, ethylene glycol, and triethylamine were purchased from Adamas Reagent, Ltd. (Shanghai, China). DMAP, I2959, is referred to Irgacure 2959 and Dichloromethane (DCM) were supplied by Aladdin Reagent Co. (Shanghai, China). Sodium hyaluronate was provided by Shanghai Yuanye Biotechnology Co. (Shanghai, China). Sodium periodate (NaIO4), F127, is referred to Pluronic F127 and ε-polylysine (EPL) were gained from Aladdin Reagent Co. (Shanghai, China). Deionized water was self-made by laboratory instruments. All materials and solvents were used as received without any further purification unless otherwise noted.

### 2.2 Methods

#### 2.2.1 Synthesis of poly (glyceryl-sebacate-acrylate) (PGSA) and preparation of PGSA nanospheres

The synthesis of poly (glycerol-sebacate) (PGS) was according to Wang’s research (Wang Y D et al., 2002), then synthesized poly (glyceryl-sebacate-acrylate) (PGSA) by modifying PGS with acryloyl chloride (Nijst et al., 2007). The structure of PGS and PGSA was verified by ^1^H nuclear magnetic resonance (^1^H-NMR) and Fourier Transform Infrared (FTIR) spectrometry. The acrylation degree of PGSA was computed from the ^1^H-NMR according to previous study (Chen et al., 2018).

The preparation method of nanospheres was as follows: At first, 30 mg PGSA was mixed in a 3 mL of ethanol and distilled water solution (V_E_: V_W_ = 1:2) to dissolve into a homogeneous solution. Photoinitiator solution was prepared by dissolving I2959 powder with deionized water, then 40 μL I2959 (2 mg/mL) was mixed with the above PGSA solution. Magnetic stirring was performed while irradiating the above solution with a UV lamp, and PGSA nanospheres (PNs) were obtained after 30 s.

#### 2.2.2 Synthesis of oxidized hyaluronic acid (OHA) and preparation of injectable hydrogels

Hyaluronic acid (HA) was oxidized to oxidized hyaluronic acid (OHA) by sodium periodate (NaIO_4_) (Hongyi et al., 2022). The structure of OHA was tested by ^1^H-NMR and FTIR spectrometry. The oxidation degree of OHA was obtained through the hydroxylamine hydrochloride method (Hongyi et al., 2022). The proportion of aldehyde groups in the OHA molecular chain, is calculated as following:

$$HA-({CHO{)}_{2}+2{NH}_{2}-OH\cdot HCl\to HA-(CH=N-OH)}_{2}+2HCl$$

$$HCl+NaOH=NaCl+H_{2}O$$

$$n=∆V\times C_{NaOH}/2$$

$$W=n\times M_{HA-CHO}+m\times M_{HA}$$

$$oxidation\;degree\left(100\%\right)=n/\left(n+m\right)=n/\left[\left(W-n\times M_{HA-CHO}\right)/M_{HA}+n\right]$$

ΔV (mL) is the volume of sodium hydroxide solution consumed when adjusting the pH of the reaction solution; n (mol) is the number of repeating units of aldehyde groups on the OHA molecular chain; W (g) is the mass of OHA; M_HA-CHO_ = 375 g/mol is the molecular weight of the OHA repeating unit; M_HA_ = 379 g/mol is the molecular weight of the hyaluronic acid repeat unit.

The preparation of hydrogels was referred to Wang’s research (Wang et al., 2019), briefly summarized as follows: OHA was freeze-dried and dissolved into an 80 mg/mL solution by using distilled water. ε-EPL was dissolved into a solution by using the same method with a concentration of 50 mg/mL and 100 mg/mL. Then F127 was dissolved into a 400 mg/mL solution under 4°C. According to the volume ratio of F127: ε-EPL: OHA as 3:0:0, 3:0:1, 3:1:1, the F127 solution and the ε-EPL solution were sequentially mixed at 4°C, and the OHA solution was added after mixing evenly, then the solution was put in a thermostatic shaker for gelation, hydrogels were named as F127, FH, FHE5, FHE10, respectively.

PNs combined with FHE10 hydrogel were prepared by the above procedure. After mixing the F127 solution and the ε-EPL solution at 4°C, PNs were dispersed in the mixing solution. Then the OHA solution was mixed with above solution at 37°C to obtain the hybrid hydrogel.

#### 2.2.3 Characterizations and testing

The microstructure of PNs was observed by SEM and TEM, then the diameter of PNs was evaluated by ImageJ. The study of particle size and z-potential was using a DLS. The injectability of PNs was tested by needle injection to observe whether it can be extruded smoothly.

Using SEM to observe the microstructure of hydrogels, and the pore size was measured *via* ImageJ. Testing whether the FHE10 can be successfully extruded from a needle proves its injectability. Phosphate buffer solution (PBS) was dropped into the test tube with hydrogels several times until the volume no longer changes and then weighed the mass. The hydrogel was lyophilized and weighed, and its water absorption ratio was computed through formula (1):

$$Water\;absorption\;ratio\left(\%\right)=\frac{{W-W}_{0}}{W_{0}}\times 100\%\;$$ (1)

When hydrogel was at swelling equilibrium, the weight was W, and when it was lyophilized, the weight was W_0_.

The prepared hydrogel was weighed and placed in an environment of 37°C with a relative humidity of 70% and then the weight of it was recorded every 1 h. The water retention ratio was computed through formula (2):

$$Water\;retention\;ratio\left(\%\right)=\frac{W_{X}-W}{W_{0}-W}\times 100\%\;$$ (2)

In the start, the weight of the hydrogel was W_0_, W_X_ was the weight of the hydrogel at the X hour, and after lyophilized the weight was W.

The free radical scavenging rate of hydrogel was measured by DPPH assay to evaluate its antioxidant capacity. The fresh 0.1 mM 1,1-diphenyl-2-trinitrophenylhydrazine (DPPH)/ethanol solution was prepared in the darkness. 30 mg of hydrogel was mixed with 3 mL of DPPH solution and cultured for 3 h and 24 h in the dark. Then a UV-Vis spectrophotometer was applied to measure the absorbance of the solution at 517 nm. The scavenging rate was computed by formula (3):

$$Scavenging\;rate\left(\%\right)=\frac{A-A_{0}}{A_{0}}\times 100\%\;$$ (3)

The absorbance of the control group was A_0_, and A was the absorbance of the samples incubated with DPPH solution after 3 and 24 h.

#### 2.2.4 Blood compatibility test *in vitro*

Blood compatibility test was carried out according to the method of GB/T 16886.4-2003 of China. The procedure is described below. 4 mL of carotid artery blood was collected from healthy SD rats, and anticoagulated whole blood was prepared using aqueous trisodium citrate solution, then freshly diluted anticoagulated whole blood was prepared using PBS buffered solution. The materials F127, FH, FHE5 and FHE10 were placed in centrifuge tubes, 3 mL of PBS solution was added to each of them, and the shaker’s temperature was set at 37°C. Then put the centrifuge tubes in the shaker, and incubated for 30 min. After 60 μL of freshly diluted anticoagulated whole blood was added to each of them, the incubation was continued for 1 h. The tubes were centrifuged at 2000 rpm/min for 5 min to get the supernatant, and then test the supernatant at 545 nm to get the absorbance value L. The same volume of distilled water and diluted anticoagulated whole blood was added to the tube as positive control group and the absorbance was tested as M. The same volume of PBS buffer solution and diluted anticoagulated whole blood was added to the tube as negative control group and the absorbance was tested as N. Three parallel samples were tested in each group. The hemolysis ratio was calculated by formula (4):

$$Hemolysis\;ratio\left(\%\right)=\frac{H_{X}-H_{N}}{H_{P}}\times 100\%\;$$ (4)

H_X_ was the absorbance of the experimental group, and H_N_, H_P_ was the absorbance of the negative and positive group respectively.

#### 2.2.5 Antibacterial performance test

*Escherichia coli* and *Staphylococcus aureus* were selected to evaluate the antibacterial performance of FHE series hydrogels. The procedure was as follows: 0.5 g of each of F127, FH, FHE5, and FHE10 hydrogels were accurately weighed in a tube and sterilized under UV for 30 min 10 μL of bacterial suspension was placed on top of the hydrogels, and after 12 h of contact, 10 mL of PBS solution was added to the tube, vortexed for 2 min, and then 10 μL of the above vortexed solution was added to the culture medium, and the bacterial solution was evenly coated on the sheep blood agar (SBA) plates using the spread-plate method and placed in a constant temperature incubator for 24 h. After the culture was completed, the antibacterial effect of the hydrogel was evaluated by observing the growth of the photographed bacteria.

#### 2.2.6 *In vitro* experiments

To assess the cytocompatibility of the materials, we selected human umbilical vein endothelial cells (HUVECs) and human foreskin fibroblasts (HFF-1). HUVECs and HFF-1 were purchased from Shanghai Cell Bank of Chinese Academy of Sciences (Shanghai, China). HUVECs were taken as an example to illustrate the culture of cells. HUVECs were grown in the culture flasks in endothelial cell medium (ECM) with 5% fetal bovine serum (FBS), 1% penicillin/streptomycin (P/S) and 1% endothelial cell growth supplement (ECGS). The flask was placed in a CO_2_ cell incubator.

The proliferation of cells co-cultured with materials was evaluated by a Cell Counting Kit-8 (CCK-8) assay. The appropriate amount of lyophilized samples F127, FH, FHE5, and FHE10 were weighed, and a solution with a concentration of 10 mg/mL by soaking in serum-free medium for 24 h were prepared. HUVECs were seeded on a 24-well plate at 1×10^4^ per well and placed in a CO_2_ cell incubator for 24 h, then the ECM medium was replaced by the ECM medium containing materials to culture cells for 1, 3, and 5 days. At a set point in time, 450 μL of ECM medium and 50 μL of the pre-warmed CCK-8 solution was placed to each well, then placed in a cell incubator for 1 h. After that, the solution was added to a 96-well plate, and then a microplate reader was used to survey the absorbance value at 450 nm wavelength.

The cytoskeletal proteins and nuclei were labeled by FITC-labeled phalloidin and 4′,6-diamidino-2-phenylindole (DAPI) respectively. The same samples were used as the CCK-8 assay. The nucleus staining was visualized under the DAPI channel (excitation = 405 nm; emission = 437-552 nm), and cytoskeletal proteins were visualized under the GFP channel (excitation = 488 nm, emission = 505-545 nm). HUVECs were planted in a 24-well plate at a density of 2×10^4^ per well, and placed in a CO_2_ cell incubator for 3 days. The cells were first washed three times with ice PBS for 15 min, and then covered with 4% cold paraformaldehyde for 15 min. For permeabilization, cells were treated with 0.1% Triton X-100 for 15 min and blocked with 1% BSA for 1 h. Finally, cells were incubated with primary antibodies at 4°C overnight. The cells were washed three times with PBS for 15 min among each step. After primary antibody incubations, cells were washed for 3 × 5 min in PBS-T (0.1% Tween 20%). Subsequently, fluorescent-labeled secondary antibody was used to incubated with cells for 2 h in dark and then DAPI was used on the cells for 15 min. All steps were washed with PBS-T. The results were observed *via* a confocal microscope.

Cell migration was tested by the transwell assay to measure the influences of materials, taking HUVECs as an example. HUVECs were planted in the upper chamber of a 24-well transwell, and 5×10^4^ cells were plated in each well. 600 μL of ECM medium containing materials were placed to the lower chamber and the ECM medium to the control group. The same samples were used as the CCK-8 assay. The culture plate was taken out after 8 h of culture and the migrated cells in upper chamber were washed with PBS for 3 times and fixed with 4% paraformaldehyde for 15 min, and stained with 0.1% crystal violet staining solution for 5 min and dried overnight. The cells were observed and taken pictures under a microscope. Counting the number of migrated cells using ImageJ.

The materials’ ability to promote HUVECs angiogenesis was tested by using Matrigel *in vitro*. 100 μL of well-mixed Matrigel was placed in the 48-well plate, then the plate was put in a cell incubator for 30 min. Each well of the plate was seeded with 3×10^4^ cells and ECM with materials was added to it, the control group was contained with ECM medium. Then the well plate was put in a cell incubator for 6 h. After this, the vascularized network structure on the surface of Matrigel was observed and photographed using a light microscope, and vessels were counted by ImageJ.

To explore the effects of materials on the gene expression of vascular endothelial growth factor (VEGF), platelet-derived growth factor (PDGF), and basic fibroblast growth factor (bFGF), HUVECs and HFF-1 were placed on 6-well plates at a density of 4 × 10 per well. After 3 days, RNA was collected from different cell samples with TRIzol reagent. qRT-PCR was then achieved with ChamQ SYBR qPCR Master Mix. The gene expression levels were normalized to GAPDH, and the expression of related genes was analyzed by the 2^−ΔΔt^ method. The sequences of target genes and internal reference genes are shown in Table 1.

**TABLE 1:** Primer sequences used for qRT-PCR.

Gene	Gene bank	Primer sequences (5′-3′)	Tm (°C)
VEGF	NCBI	F: AGG​GCA​GAA​TCA​TCA​CGA​AGT	61.2
	geneID7422	R: AGG​GTC​TCG​ATT​GGA​TGG​CA	62.9
PDGF	NCBI	F: CTC​GAT​CCG​CTC​CTT​TGA​TGA	61.7
	geneID5155	R: CGT​TGG​TGC​GGT​CTA​TGA​G	60.2
bFGF	NCBI	F: AGA​AGA​GCG​ACC​CTC​ACA​TCA	62.7
	geneID2247	R: CGG​TTA​GCA​CAC​ACT​CCT​TTG	61.2
GAPDH	NCBI	F: ACA​ACT​TTG​GTA​TCG​TGG​AAG​G	62.1
	geneID2597	R: GCC​ATC​ACG​CCA​CAG​TTT​C	61.1

#### 2.1.7 *In vivo* experiments

All animal experiments were authorized by the Animal Research Ethics Committee of the First Affiliated Hospital of Zhejiang University and were performed by the National Institutes of Health Guidelines for the Care and Use of Laboratory Animals. Forty healthy rats were randomly selected as experimental rats and divided into four groups, which were marked as the control group, PNs group, FHE10 group, and FHE10@PNs group. A rat skin defect model was established based on previous research methods (Wang et al., 2013). Each rat was weighed and 4%Pentobarbital (40 mg/kg) was used to anesthetize by intraperitoneal administration. After the injection, the vital signs of the rat were noted. Two symmetrical circular marks with a diameter of 2 cm were made on the depilation area on the back of the rat, and the full-thickness skin was cut off. The bleeding was compressed by the gauze until there was no obvious bleeding. In the next step, the wound was covered with materials and wrapped with gauze. After the operation, penicillin was injected to prevent infection, and the health of the rats was closely observed. Rats in each group were sacrificed 7 and 14 days after surgery, and the skin tissues at the edge of the wound were collected for immunohistochemical analysis.

The healing of skin wounds on days 0, 3, 7, and 14 after the operation was recorded by a camera, and the area of the wound was calculated by ImageJ. The wound healing ratio was computed by formula (5):

$$Wound\;healing\;ratio\left(\%\right)=\frac{S_{0}-S}{S_{0}}\times 100\%\;$$ (5)

Wound area on day 0 was represented by S_0_, and the wound area on days 3, 7, and 14 was represented by S.

The skin tissues were completely immersed in 10% formalin solution overnight, then in graded alcohol (75%, 85%, 95%, 100%) for dehydrating. Paraffin was used to embedded the cleared tissues, and the tissue block was cooled overnight then cut into 5 μm of sections.

The regeneration of wound *epidermis* was assessed by staining the tissue sections with hematoxylin and eosin (H&E). H&E staining was carried out according to previous study (Yoon et al., 2016). For H&E staining, paraffin-embedded sections were first deparaffinized and hydrate to water. Then, nucleus was stained with Hematoxylin Solution (Solarbio, China) for 15 min followed by rinsing in running tap water. Next, the tissue section was differentiated by Differentiation Solution (Solarbio, China) for 3 min and re-dyed with Eosin Y Aqueous Solution (Solarbio, China) for 1 min. After the sections were dehydrated and sealed, photos were taken and analyzed. The wound reepithelialization ratio was calculated using formula (6):

$$Reepithelialization\;ratio\left(\%\right)=\frac{L_{0}-L}{L_{0}}\times 100\%\;$$ (6)

L_0_ represents the original wound length, and L represents the incompletely healed wound *epidermis* length.

Masson’s trichrome staining was accomplished on tissue sections to evaluate the collagen deposition. Masson’s trichome staining were carried out according to previous study (Zhao et al., 2017). For Masson’s trichome stain, a Masson trichrome staining kit (Sigma-Aldrich, America) was used to stain the tissue section according to the manufacturer’s protocol. To observe and photograph the stained sections with an optical microscope. The area of the blue part was counted and quantitatively analyzed using ImageJ to evaluate the collagen deposition rate in the new tissue.

CD31 immunohistochemical staining and CD31/α-SMA immunofluorescence staining were performed on tissue sections to evaluate angiogenesis in wound tissue. CD31 immunohistochemical staining were carried out according to previous study (Kong et al., 2018). As for CD31immunohistochemical staining: A two-step immunohistochemistry kit (Zhongshan Golden Bridge Biotechnology, China) was used for this study according to the manufacturer’s protocol. To observe and photograph the stained sections with a light microscope, and two pathologists were asked to count the number of blood vessels in the stained sections under double-blind conditions.

#### 2.1.8 Statistical analysis

Three parallel samples were prepared for the experiments, and the mean ± standard deviation was employed as the analysis data. One-way ANOVA was applied to process statistical differences between groups. When *p* < 0.05 (*) *p* < 0.01 (**) the differences had statistical significance.

## 3 Results and discussions

### 3.1 Characterization of PNs and FHE hydrogel

**FIGURE 1:**
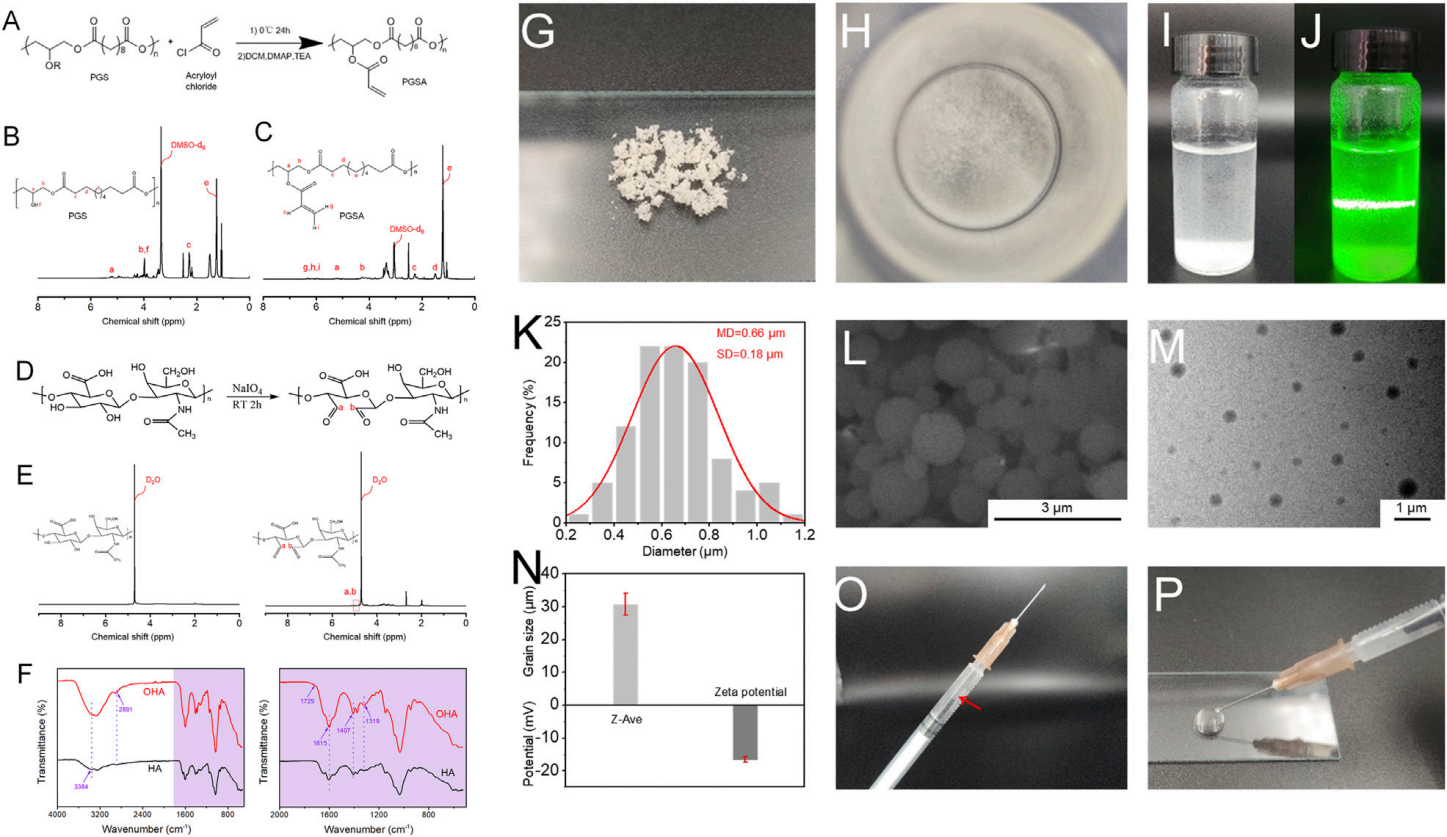
**(A)** The reaction mechanism route of PGSA; **(B)** ^1^H-NMR spectrum of PGS; **(C)** ^1^H-NMR spectrum of PGSA. **(D)** The reaction mechanism route of OHA; **(E)** ^1^H-NMR spectrum of HA and OHA; **(F)** FTIR spectrum of HA and OHA. **(G)** Apparent morphology of PNs, as revealed by digital images; **(H)** Digital photographs of PNs dispersed in deionized water; **(I)** and **(J)** PNs were uniformly dispersed in deionized water under natural light, and Tyndall effect appeared after beam irradiation; **(K)** The diameter distribution histograms of PNs; **(L)** SEM image of PNs; **(M)** TEM image of PNs; **(N)** Particle size and potential of PNs; **(O)** and **(P)** Schematic illustration of the injectability of PNs. The red arrow means PNs were uniformly dispersed in the syringe.

PGS and PGSA were successfully synthesized (Figures 1A–C and Supplementay Figure S1). The ^1^H-NMR analysis indicates that the degree of acylation was 32%. OHA was obtained by modification (Figures 1D–F). The oxidation degree of OHA was 12.5% by the method of hydroxylamine hydrochloride (Wang et al., 2019). In the presence of photoinitiator, the aqueous ethanol solution of PGSA was subjected to ultraviolet irradiation and magnetic stirring to form nanospheres PNs with uniform particle size.

Figures 1G, H showed the state of the PNs under dry conditions and the state of dispersion in water, respectively. The PNs nanospheres are easy to aggregate together in the dry state, which may be caused by their viscosity. When it was put in deionized water, it can be evenly dispersed. Using a beam to irradiate the PNs solution, the Tyndall effect can be observed from Figures 1I, J, indicating that the nanospheres were uniformly dispersed. It can be seen from the SEM image (Figure 1L) that PNs have a relatively smooth surface. Through ImageJ statistical calculation, it can be obtained that the particle size distribution of PNs (Figure 1K) is in the range of 0.2–1.2 μm, mainly in the range of 0.6–0.8 μm, which is relatively uniform. The dispersion of PNs can be seen from the transmission electron microscopy (TEM) image (Figure 1M). There is a certain difference between the particle size range measured by the dynamic light scattering (DLS) (Figure 1N) instrument and the results of scanning electron microscope (SEM). It is speculated that during the test process, the PNs aggregated due to the sedimentation of their weight, and the measured particle size reached several tens of microns. The surface charge of PNs is negative (Figure 1N). The extrusion experiment of a 1 mL syringe with a needle size of 0.45 × 16 mm proved that PNs could be extruded without sticking to the needle tube (Figures 1O, P). As can be seen the white PNs are evenly dispersed in the syringe and do not adhere to the wall. By extruding on the glass slide, PNs have good injectability and will not block the needle tube.

**FIGURE 2:**
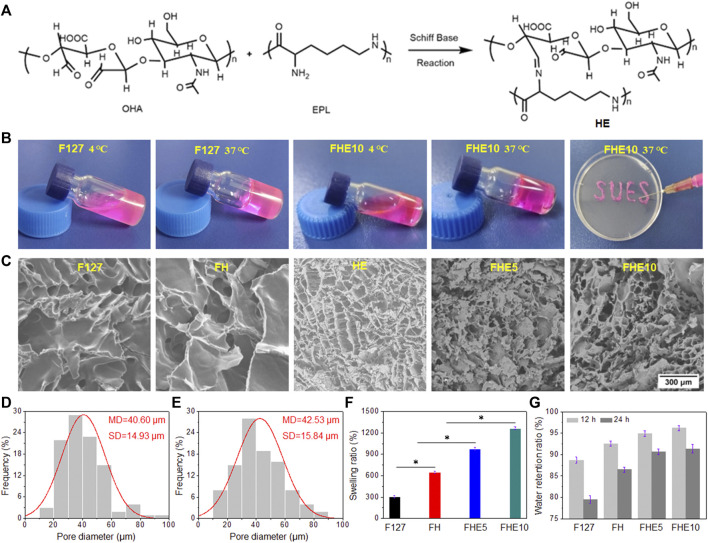
**(A)** Schiff base reaction mechanism route; **(B)** Photographs of F127 and FHE10 hydrogel before and after gelation, and FHE10 proved its injectability by extruding SUES; **(C)**The SEM images of F127, FH, HE, FHE5 and FHE10 hydrogel; **(D)** and **(E)** The pore diameter distribution of FHE5 and FHE10, respectively; **(F)** The swelling ratio of F127, FH, FHE5, and FHE10 hydrogel; **(G)** The water retention ratio of F127, FH, FHE5, and FHE10 hydrogel.

F127 is a temperature-sensitive material. At low temperature, it is in a solution state, and when the temperature is raised, it transforms into a gel state. After mixing F127 with OHA and EPL, the whole solution has temperature sensitive properties. When the temperature was at 4°C, the mixed solution became a sol state, and when the temperature was raised up to 37°C, it became a gel, so it belonged to a thermosensitive gel (Figure 2B). After the gel was loaded into the syringe, it was slowly extruded into letters such as “SUES” (Figure 2B), and the extrusion was smooth without clogging, which proved that the hydrogel system has good injectable performance. It can be seen from Figure 2C that F127 and FH hydrogel had no pores, while FHE hydrogel had pores. The measurement of pore diameter was by ImageJ, and Figures 2D, E were the pore size of FHE5 and FHE10, respectively. Figure 2F is the swelling ratio of hydrogels. The swelling ratio of FHE5 and FHE10 hydrogel increased significantly, which may be due to the reticular pore structure formed after the chemical reaction, thereby improving the water absorption, and swelling ability of the hydrogel. Due to the prolongation of time, and the inevitable loss of water, the FHE5 and FHE10 hydrogel has a stronger water-locking ability (Figure 2G).

**FIGURE 3:**
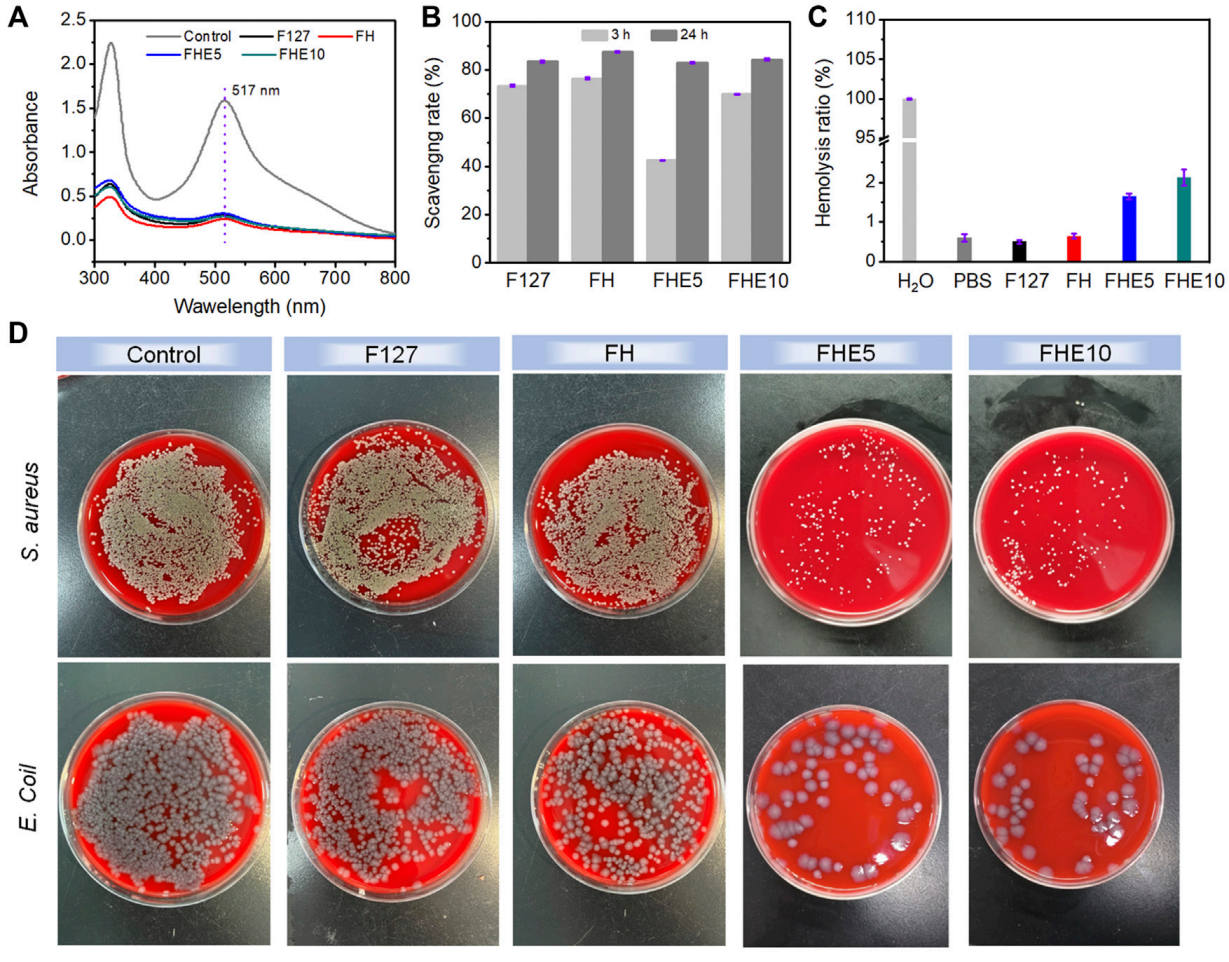
**(A)** UV absorption of DPPH solution after co-incubation with F127, FH, FHE5, and FHE10 hydrogel; **(B)** Scavenging rate of F127, FH, FHE5, and FHE10 hydrogel; **(C)** Hemolysis ratio of F127, FH, FHE5, and FHE10 hydrogel; **(D)** Photographs of survival bacteria clones on SBA plates after coculturing with F127, FH, FHE5, and FHE10 hydrogel.

A UV spectrophotometer was used to measure the absorbance of the mixed solution, the UV absorption of the solution after co-incubation with the material decreased (Figure 3A) and the color faded (Supplementary Figure S2) compared with the control group. The scavenging rate of F127, FH, FHE5, and FHE10 hydrogel reached more than 80% after 24 h (Figure 3B), indicating that the materials have good antioxidant properties. The ability to scavenge free radicals should come from many hydroxyl groups in the F127 molecule. In addition, the molecular chain of OHA also contains many hydroxyl groups, so the hydrogel has a strong ability to scavenge free radicals.

Supplementary Figure S2 showed that except for the control group, the centrifuge tubes containing the material were centrifuged after incubation, the red blood cells were deposited at the bottom without breaking, the supernatant was clarified, and the hemolysis ratio was less than 5% (Figure 3C), proving that the material did not cause hemolysis. The prepared hydrogels, only FHE5 and FHE10 have certain antibacterial properties (Figure 3D). Judging from the growth of bacteria in the control group and the F127 and FH groups, the bacterial colonies on the medium grew well, and it was speculated that the pure F127 hydrogel and FH hydrogel did not have antibacterial effects. Comparing the FHE5 and FHE10 groups, the growth of bacteria is inhibited, which is attributed to the fact that ε-EPL itself has a certain antibacterial activity, and the protonated amino group can damage the cell wall of bacteria and lead to bacterial death.

In summary, FHE10 hydrogel and PNs were selected for follow-up studies.

### 3.2 Effects of PNs, FHE10, and FHE10@PNs on proliferation, morphology, migration, the tube formation, and angiogenesis-related gene expression *in vitro*

**FIGURE 4:**
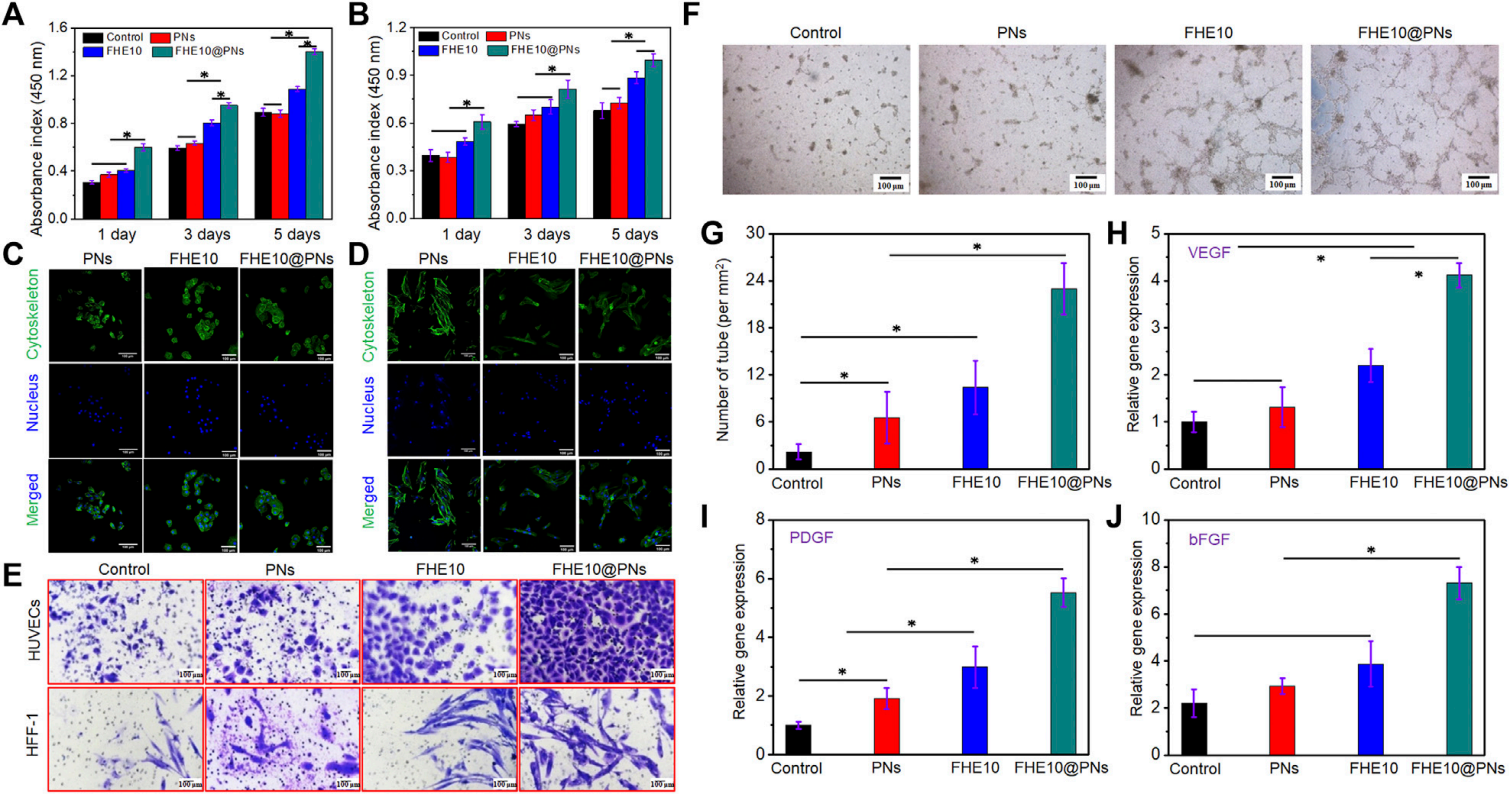
**(A,B)** CCK-8 assay indicated that proliferation of HUVECs and HFF-1 after culturing with PNs, FHE10, and FHE10@PNs, respectively; **(C,D)** DAPI (blue)/FITC-phalloidin (green) staining assay of HUVECs and HFF-1 with PNs, FHE10, and FHE10@PNs after culturing, respec-tively; **(E)** Transwell assay showed that migration of HUVECs and HFF-1 were stained by crystal violet; **(F)** Tube formation assay; **(G)** Quantitative analysis of tube formation; **(H–J)** qRT-PCR analysis of VEGF, PDGF, and bFGF.

HUVECs and HFF-1 in good growth state were cultured by using the material extract. The FHE10@PNs promoted cell proliferation more than PNs or FHE10 alone, and with the increase of culture time, cell proliferation gradually increased, indicating that cells also adapted to the surrounding environment and achieved the best growth state (Figures 4A, B).

HUVECs and HFF-1 were cultured for 3 days, then their morphology was observed by a light microscope (Figures 4C, D). HUVECs cells in experiment group have no significant change in cell morphology when compared to the control group. The cell body of HFF-1 was fusiform or irregular triangular, with an oval nucleus in the center and a cytoplasmic protrusion, which was radial when growing. The characteristics were consistent with the normal proliferation of HUVECs and HFF-1.

The migration of HUVECs and HFF-1 co-cultured with the material was observed by a transwell migration assay (Figure 4E). The groups added with materials all could promote cell migration, and the FHE10@PNs had the strongest ability to promote cell migration, so the combined action of the two had a synergistic effect on cell migration. By a quantitative analysis of the number of migrating cells (Supplementary Figure S3), the results were consistent with the staining, which proved that the materials indeed had the chemotactic attraction to cells.

*In vitro* tube formation assay was performed by co-cultured PNs, FHE10, and FHE10@PNs with HUVECs to evaluate the tube formation ability (Figures 4F, G). HUVECs co-cultured with each group and formed a network of capillary-like structures on the Matrigel after 6 h, and each group had some differences. The tube’s diameter is shown in the supporting information (Supplementary Figure S4). Compared with the control group, the PNs, FHE10, and FHE10@PNs groups all could promote angiogenesis, and the FHE10@PNs group had the best-promoting effects.

The effects of PNs, FHE10, and FHE10@PNs on the expression of angiogenesis-related genes after co-culture with HUVECs and HFF-1 for 7 days were evaluated (Figures 4H–J). The gene expression levels of VEGF, PDGF, bFGF in the PNs, FHE10, and FHE10@PNs groups were better than those in the control group, while the FHE10@PNs group had the best gene expression levels.

### 3.3 *In vivo* wound tissue-repair ability

**FIGURE 5:**
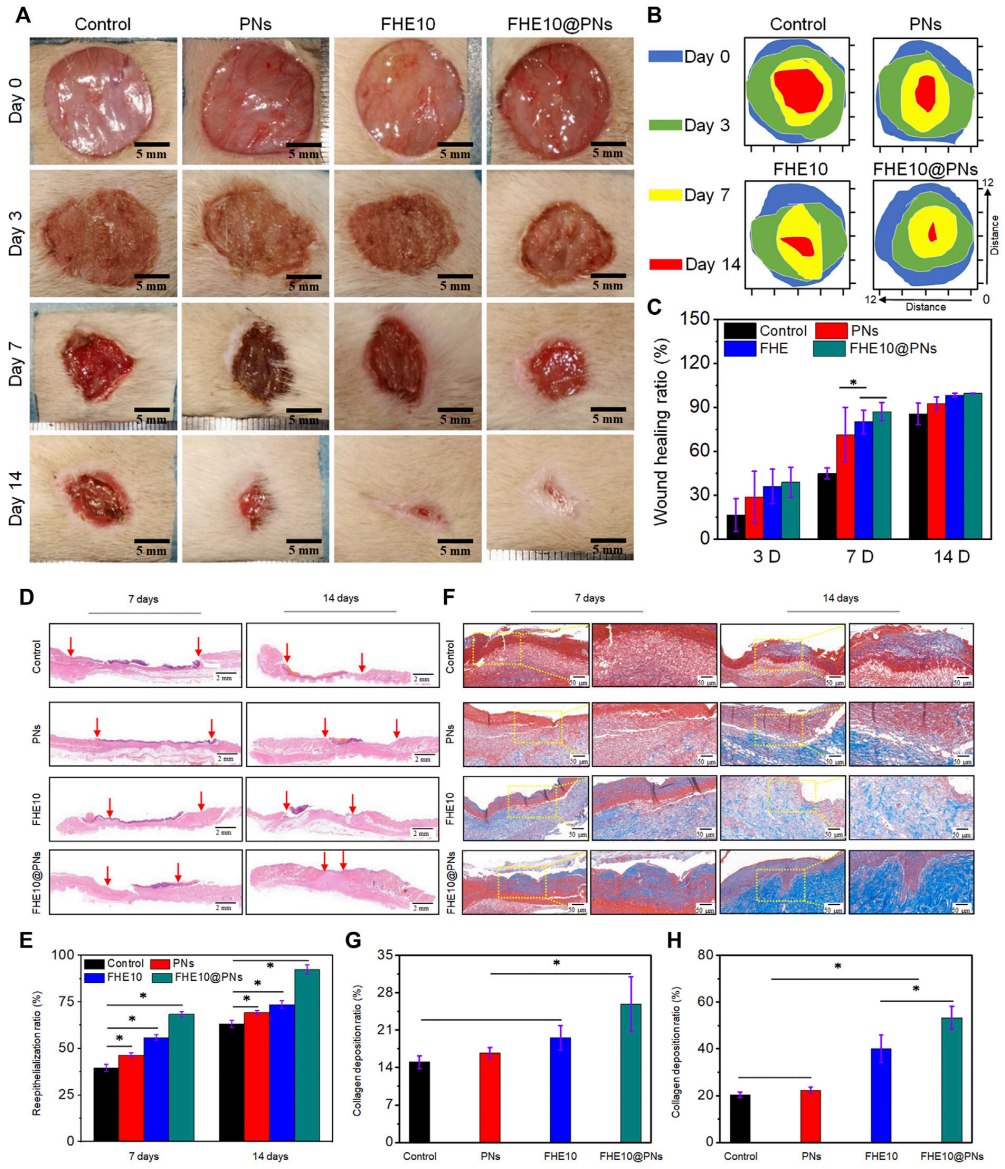
**(A)** Wound images of control, PNs, FHE10, FHE10@PNs at 0, 3, 7 and 14 days post-operation; **(B)**Traces of wound repair for each treatment group *in vivo*. Blue, green, yellow, and red areas correspond to the wound area at n (*n* = 0, 3, 7 and 14) days, respectively; **(C)** Wound healing ratio of the defects treated with saline (control), PNs, FHE10, FHE10@PNs at 0, 3, 7 and 14 days post-operation; **(D)** H&E stained images; **(E)** The reepithelialization rate of wound defect at 7 and 14 days after treatment with saline (control), PNs, FHE10, and FHE10@PNs; **(F)** Masson’s trichrome stained images; **(G,H)**The collagen deposition in the wound sites was evaluated by ImageJ.

Encouraged by the controllable injectability, favorable biocompatibility and antibacterial activity, outstanding pro-angiogenic properties of the prepared hydrogels revealed above, FHE10@PNs was chosen and further used as a potential wound dressing for skin repair to evaluate the *in vivo* wound healing performance. Figure 5A visually showed the wound healing progress treated by the saline, PNs, FHE10, and FHE10@PNs for 0, 3, 7, and 14 days, respectively. The wound healing rates of the control, PNs, FHE10, and FHE10@PNs groups were measured by using ImageJ at each observation time point, and the FHE10@PNs group showed the best performance in wound healing rate, as compared with the other three groups with a significant difference at Day 7 (*p* < 0.05). The average wound healing rates of control, PNs, FHE10, and FHE10@PNs were 44.8%, 71.3%, 80.0%, and 87.1%, respectively. The continuous observation for up to 14 days after operation showed unhealed wounds in the control, PNs, and FHE10 groups, which almost completely healed in the FHE10@PNs group with a wound healing rate of 99.5% (Figures 5A–C).

We further clarified the quality of regenerated wound tissue by using H&E staining and Masson trichrome staining at the observation time points of day 7 and day 14 (Figures 5D, F). FHE10@PNs exhibited better healing effects than the other three groups (Figures 5D, E). After 7 days of the skin defect, the epidermal regeneration rates of the control group, PNs group, FHE group, and FHE10@PNs group reached 39.5%, 46.3%, 55.8%, and 68.3%, respectively. After 14 days of treatment, the wounds treated with FHE10@PNs exhibited almost no open wounds with a healing rate of 92.3% and become smooth with new epidermal tissue. In contrast, most of the wounds in the other groups remained significantly more open and the scars were uneven.

Collagen fibers are a key component related to granulation tissue and the dermal extracellular matrix during the wound healing processes (Diller and Tabor, 2022). Thus, Masson staining was performed to evaluate the deposition and organization of collagen fibers in regenerative skin tissue at 7 and 14 days post-surgery (Figures 5F, G). As presented in Figure 5G, in terms of the area of collagen deposition, a significant difference was found across the groups at 7 days (15.05%, 16.71%, 19.61%, 25.89% for control, PNs, FHE10, FHE10@PNs, respectively). On day 14, mature collagen fibers are observed in the FHE10@PNs group and the collagen deposition is denser and more organized (Figure 5H). On the other hand, the collagen fibers presented in the other groups are still partially dysplastic.

### 3.4 FHE10@PNs hydrogel facilitates angiogenesis

**FIGURE 6:**
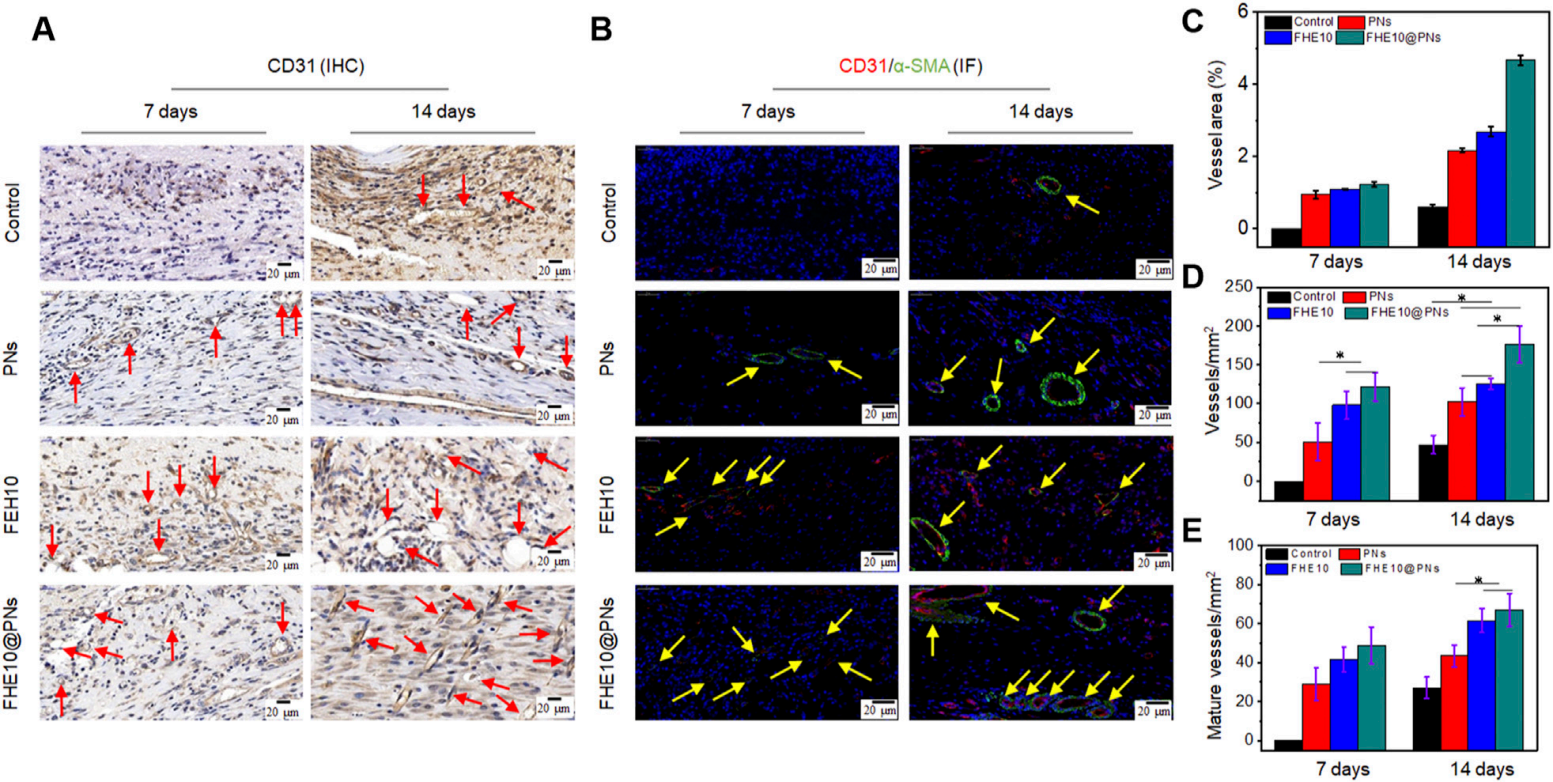
IHC and IF analysis of neovascularization in wound of control, PNs, FHE10, and FHE10@PNs groups, respectively. **(A)** IHC staining for CD31, red arrows mean newly formed blood vessels; **(B)** IF co-staining for α-SMA and CD31, yellow arrows mean mature vessels; **(C)** Area analysis of mature blood vessels at 7 and 14 days post-operation; **(D)** Quantitative analysis of newly formed blood vessels at 7 and 14 days post-operation; **(E)** Quantitative analysis of mature vessels at 7 and 14 days post-operation.

The angiogenesis is essential for nutrient and oxygen supply in the process of skin wound repair. To better elucidate the properties of the FHE10@PNs hydrogel on angiogenesis, CD31 and α-SMA were selected for immunohistochemical staining analysis. As plotted in Figures 6A, D, the FHE10@PNs group exhibited significantly increased neovascularization based on a high level of CD31 expression on both day 7 and day 14. In contrast, either PNs or FHE10 alone displayed less CD31 expression, confirming that the FHE10@PNs hydrogels provided a favorable 3D microenvironment scaffold for blood vessel formation.

Moreover, the colocalization of α-SMA and CD31 was seen as a biomarker of a normalized vessel (Wu et al., 2020). Positively stained endothelial cells and smooth muscle cells lining the abundant vessel networks were observed in the skin regenerated tissue treated with FHE10@PNs, but significantly, less were observed in the control or other material-treated groups. The mature vessel areas on day 7 and day 14 was significantly bigger than the other groups (Figures 6B,C,E). On the basis of the above results, FHE10@PNs hydrogels not only showed the ability to facilitate neovascularization but also to promote subsequent vascular maturation in the process of skin wound repair.

## 4 Conclusion

In this study, a hybrid hydrogel was prepared by preparing PGS acrylate derivative photocurable nanospheres and mixing them with injectable oxidized hyaluronic acid hydrogel FHE10. The *in vitro* results showed that the FHE10@PNs group could up-regulate the proliferation, migration, and expression of genes related to angiogenesis of HUVECs and HFF-1. *In vivo* studies further demonstrated that the prepared FHE10@PNs group significantly promoted wound reepithelialization, collagen deposition, and neovascularization, and finally promoted wound healing. Therefore, the preparation of injectable FHE10@PNs has better effects on promoting reepithelialization, collagen deposition, angiogenesis and wound healing than using PNs or FHE10 hydrogel alone. In conclusion, the FHE@PNs may be a potential material to promote reepithelialization, collagen deposition, and angiogenesis in wound healing.

## Data Availability

The original contributions presented in the study are included in the article/Supplementary Material, further inquiries can be directed to the corresponding authors.
