# Supplementary material for: A poly (glycerol-sebacate-acrylate) nanosphere enhanced injectable hydrogel for wound treatment
Source: Front Bioeng Biotechnol. 2023 Jan 12;10:1091122. doi: 10.3389/fbioe.2022.1091122 (PMC9877222; doi:10.3389/fbioe.2022.1091122)
Supplement: Supplementary file 1 [file DataSheet1.pdf]

Supplementary Material

1 FTIR Analysis of Pre-PGS and PGSA

It can be seen from the FTIR that a new peak is generated, which is attributed to the carbon-carbon double bond, which means that the acryloyl chloride is successfully grafted to the side chain of PGS.

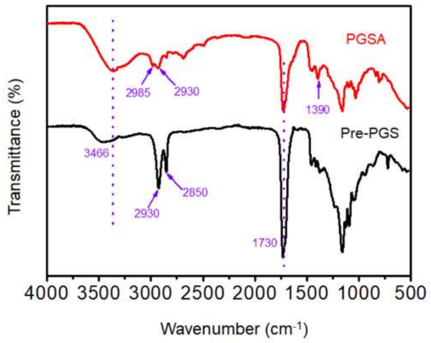

Supplementary Figure 2. FTIR of Pre-PGS and PGSA.

2 Pictures of DPPH free radical scavenging and hemolysis experiment

As can be seen from Figures S2A and S2B, after the material was co-incubated with DPPH solution for 3h and 24h, the color faded, and free radical scavenging occurred.

It can be seen from Figure S2C that after materials and blood were co-cultured and centrifuged, the red blood cells of the positive control group were ruptured, causing the solution turn to red. Other groups were clear and transparent, and the red blood cells were deposited at the bottom, which means that the material did not cause hemolysis.

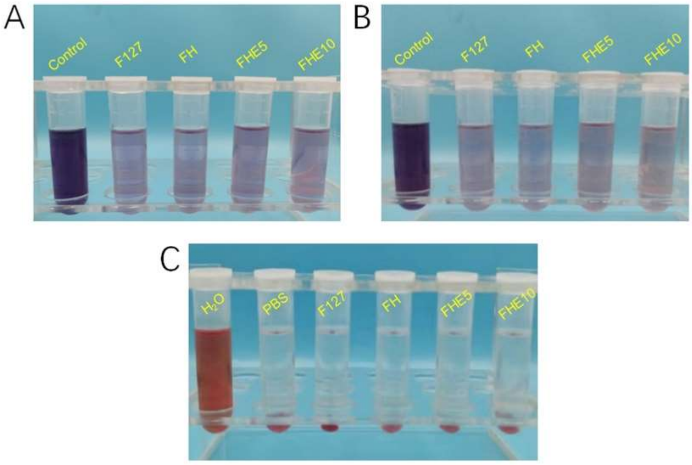

Supplementary Figure 2. (A) and (B) Picture of the material after co-culture with DPPH; (C) Picture of hemolysis experiment.

3 Quantitative analysis of HUVECs and HFF-1 migration.

It can be seen from the figure that the number of cells migrated in the control group was the least, and the number of cells migrated in the PNs group, the FHE10 group, and the PNs+FHE10 group increased in turn, especially in the PNs+FHE10 group, which proved that the co-use of the two materials has a synergistic effect on cell migration.

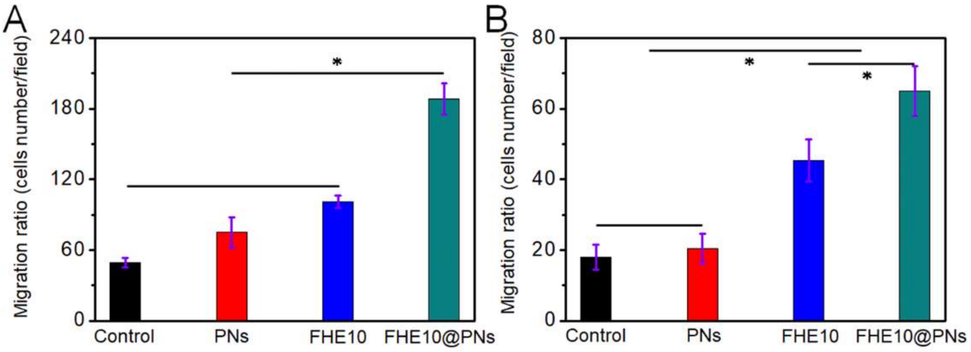

Supplementary Figure 3. Quantitative analysis of HUVECs and HFF-1 migration. (A) The migration ratio of HUVECs; (B) The migration ratio of HFF-1.

4 Quantitative analysis of tube diameter.

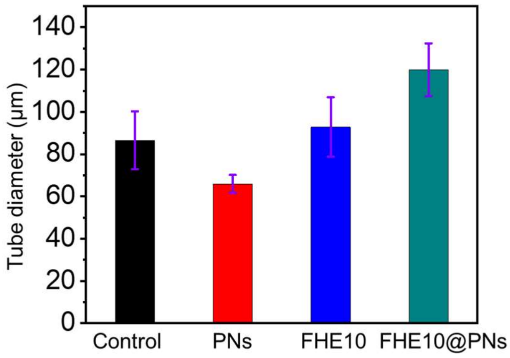

Supplementary Figure 4. Quantitative analysis of tube diameter.
